# Triphalangeal thump, thumb duplication, and syndactyly: The first case report in the literature

**DOI:** 10.1097/MD.0000000000031237

**Published:** 2022-10-21

**Authors:** Sarya Swed, Abdulqadir J. Nashwan, Hiba Haj Saleh, Yamane Chawa, Alaa Baria, Aladdin Etr

**Affiliations:** a Faculty of Medicine, Aleppo University, Aleppo, Syria; b Nursing Department, Hamad Medical Corporation, Doha, Qatar; c Department of Endocrinology, Aleppo University Hospital, Aleppo, Syria; d Department of Plastic Surgery, Aleppo University Hospital, Aleppo, Syria.

**Keywords:** case report, syndactyly, thumb duplication, triphalangeal thump

## Abstract

Triphalangeal thumb (TPT) is a rare congenital malformation where the thumb has three phalanges instead of two. Syndactyly is a condition in which children are born with fused or webbed fingers. The combination of TPT, Syndactyly, and thumb duplication is extremely rare, especially when these deformities are combined in one hand.

**Patient concerns:**

Hand abnormalities and polydactyl have been reported in a 1-year-old boy.

**Diagnosis:**

A clinical examination reveals two thumb duplications, finger fusion (Syndactyly), and a thumb with three phalanges (TPT). The diagnosis was based on clinical findings and an X-ray image of the hand.

**Interventions:**

The Z-plasty method was used to remove the adhesion between the thumb and forefinger, as well as the removal of the medial and distal phalanx of the thumb’s medial tip.

**Outcomes:**

The patient was followed for 2 months and found him in good health. To authors’ knowledge, we described an unusual case from Syria, considered the first in medical history.

**Lessons Learned:**

General and plastic surgeons should be aware about this unusual mix of the three abnormalities. The family history must also be carefully investigated to explore the occurrence of hereditary illnesses.

## 1. Introduction

Transverse deficiencies, longitudinal deficiencies of the forearm, deficiencies of the hands and fingers, and other complicated abnormalities of the upper limbs are all caused by congenital malformations. Usually, they are identified during a 20-week anatomic ultrasound. Triphalangeal thumb (TPT), historically first recorded by Columbo in 1559,^[1]^ is a congenital hand deformity in which the thumb possesses an additional phalanx. Despite having an incidence of 1 in 25,000 neonates, TPT is an uncommon abnormality, but its diverse phenotypic characteristics are frequent.^[2]^ It is inherited autosomal dominantly and is connected to LMBR1 on chr7q36.3. Additional deformities include Syndactyly, Ulnar Polydactyly, Cleft Hand and Radial Dysplasia, Differences in the Lower Extremities, and as part of a syndrome involving abnormalities in one or more other systems.^[3]^ Aase syndrome, which is likely a subtype of Diamond-Blackfan anemia, is distinguished by having TPTs.^[4]^ TPT may be categorized according to the three various forms of the additional phalanx (wedge, trapezoidal, or rectangular) as defined by Wood,^[5]^ or it can be classified according to Wassel^[6]^ when radial polydactyly is present. The Duplication thumb is another congenital abnormality.

Given that it affects 1 in every 3000 newborns, it is one of the most prevalent congenital hand deformities. Polydactyly, including duplication thumb, may affect the hands or the feet. The fusing of neighboring digits is the last abnormality, known as Syndactyly. Of all congenital hand abnormalities, it is the most prevalent, occurring in around 1 in 2000 births. This case report shows a very rare correlation between 3 deformities:TPT, thumb duplication, and Syndactyly. The appearance is the main reason parents come to the outpatient clinic. Surgical treatment for the various anomalies improves the shape and makes it more functional.

## 2. Case presentation

**Figure 1.**
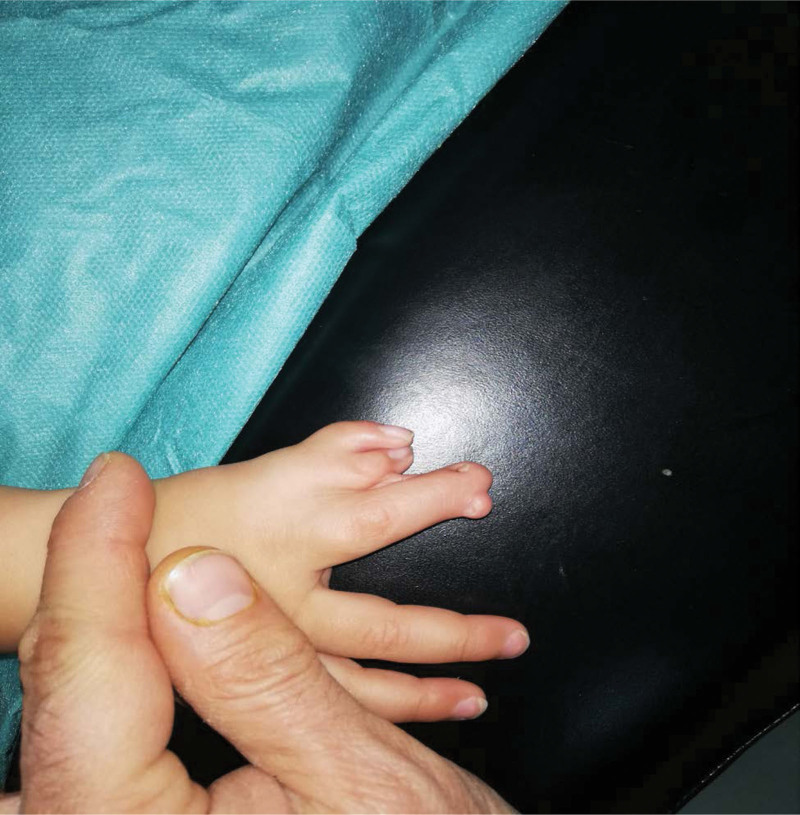
The clinical examination of the right hand that shows the deformities.

**Figure 2.**
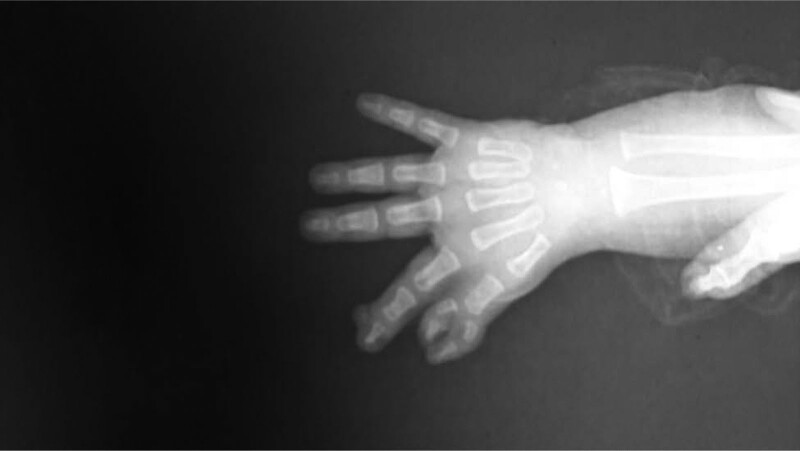
The picture X-rays of right hand that shows the deformities (triphalangeal thump, thumb duplication and syndactyly).

**Figure 3.**
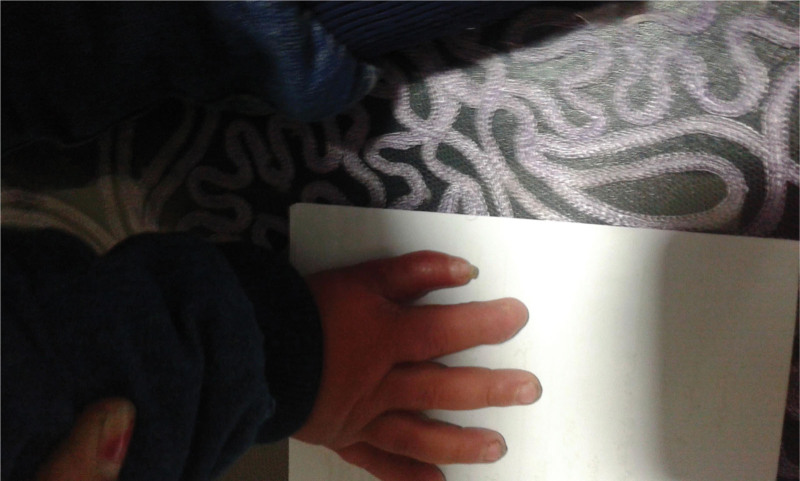
The picture of the right hand after following-up the patient that shows there is no any complication with improving the shape of hand’s patient.

A 1-year-old male was admitted to our hospital complaining of the presence of hand deformities and polydactyly. There is no family, medical, surgical or allergic history. Two thumbs were observed (thumb duplication), a fusion of fingers with each other (Syndactyly), and a thumb containing three phalanges (TPT) (Fig. 1), and the clinical examination of the rest of the body was normal. And this malformation was isolated and was not associated with any other malformation. The study of hand X-rays revealed abnormal hand bone tissue structure (Fig. 2). We did laboratory tests such as complete blood count, hemoglobin, and electrolytes, which were normal. We were unable to perform any genetic tests due to poor financial capabilities. After consulting with a pediatrician, the decision about surgery was taken, the operation was performed under general anesthesia, and it took about 90 min. The surgical procedure was performed by excision of the extra distal and medial phalanges from the medial side of the double TPT and the dissociation of the fusion by making a surgical incision with the technique of Z-plasty at the site of the excision and a web was made between the thumb and forefinger. The Kirschner wire (pinhead) was placed straight in the lateral side of the remaining thumb and the extra sesamoid bone excision in the forefinger. The operation was successful. After the operation, the child was given an analgesic drug (Ibuprofen 100 mg syrup) and a prophylaxis antibiotic (Cefixime 100 mg syrup) to reduce pain and infection risk. He was discharged from the hospital after 3 days. The child was followed up by changing the bandage every 2 days with the use of povidone-iodine and Fucidin ointment, and the stitches were removed after 15 days, and the Kirschner wire was removed after a month. Then, the child was subjected to physiotherapy. The follow-up of the child continued for 2 months, and there was difficulty in evaluating the final result of the surgical procedure due to the age of the young child, but the movement of the hand after the operation was normal, and the result is generally good (Fig. 3).

## 3. Discussion

We present the condition of thumb duplication with the TPT with clinodactyly and Syndactyly. TPT is rare, and the incidence rate is 1/25,000 live births. It is a congenital malformation in which the thumb contains 3 phalanges instead of 2. TPT may be a part of a syndrome like Blackfan anemia and may be autosomal dominant in ratio 2/3. It is associated with more malformation like polydactyly syndactyly claw-like hand or foot. The majority of the abnormalities are found on chromosome 7q3b, along with the added phalanx, which may have a variety of forms, including wedge or rectangular, from the distal interphalangeal joint to the radiocarpal joint, as well as joints, ligaments, muscles, and tendons of the first ray that can be hypoplastic deformed or missing with variable degrees of stiffness or instability. Classifying TPT depends on wood and Buck Gramcko, which describe three different shapes of the extra phalanx, while Buck Gramcko describes different encountered shapes from small to large.^[7]^ In addition, the International Federation of Societies for Surgery of the Hand Committee recommended Swanson’s classification of Congenital cases as a proper system for use by hand and upper limb surgeons working in this field which presented an understanding of embryological limb development, and it has also distinguished effective and useful.^[8]^ On the other side, Syndactyly forms one of the most frequent inheritance limb deformities.^[9]^ The incidence rate of Syndactyly ranges from 1.1/10,000 in the northern Netherlands^[10]^ to 1.3/10,000 in New York state because of geographical and registry differences,^[11]^ and in China is 7.4/10,000 in 1998–2009.^[12]^ Thumb duplication is another congenital polydactyly disorder when a kid is born with more than five digits on their hands and feet. Both the hands and the feet may be affected.

Thumb duplication is the most frequent congenital upper limb abnormality. In addition, preaxial polydactyly is often classified under this heading. The importance of our case comes because it is the first case in medical literature and because we report the association of these three conditions together. The patient suffered from the TPT with clinodactyly and Syndactyly. The wood classification was 5, and Buck-Gramcko was 7. An X-ray confirmed the diagnosis. Surgical treatment was chosen to remove deformities, reduce the length of the thumb, and create a good function with the observation other limb is also triphalangeal. The condition has been followed up for two months, and the patient’s condition was good without complications after surgery.

## 4. Conclusion

In this case report, we provide the first instance in the medical literature where three anomalies are present at the same time. This uncommon combination of these defects will give plastic surgeons a distinctive personality. When confronted with a situation like this, they need to ensure that an exact therapy, such as surgery fixing, is carried out and then monitor the results.

## Author contributions

All authors read and approved the final manuscript.

**Conceptualization:** Sarya Swed.

**Writing – original draft:** Sarya Swed, Abdulqadir J. Nashwan, Hiba Haj Saleh, Yamane Chawa, Alaa Baria, Aladdin Etr.

**Writing – review & editing:** Sarya Swed, Abdulqadir J. Nashwan, Hiba Haj Saleh, Yamane Chawa, Alaa Baria, Aladdin Etr.

## Acknowledgments

We would like to thank the patient’s parents for helping us to report this rare case in the literature.
